# ParticleCall: A particle filter for base calling in next-generation sequencing systems

**DOI:** 10.1186/1471-2105-13-160

**Published:** 2012-07-09

**Authors:** Xiaohu Shen, Haris Vikalo

**Affiliations:** 1 Department of Electrical and Computer Engineering, University of Texas at Austin, 1 University Station C0803, TX, 78712-0240, US

## Abstract

**Background:**

Next-generation sequencing systems are capable of rapid and cost-effective DNA sequencing, thus enabling routine sequencing tasks and taking us one step closer to personalized medicine. Accuracy and lengths of their reads, however, are yet to surpass those provided by the conventional Sanger sequencing method. This motivates the search for computationally efficient algorithms capable of reliable and accurate detection of the order of nucleotides in short DNA fragments from the acquired data.

**Results:**

In this paper, we consider Illumina’s sequencing-by-synthesis platform which relies on reversible terminator chemistry and describe the acquired signal by reformulating its mathematical model as a Hidden Markov Model. Relying on this model and sequential Monte Carlo methods, we develop a parameter estimation and base calling scheme called ParticleCall. ParticleCall is tested on a data set obtained by sequencing phiX174 bacteriophage using Illumina’s Genome Analyzer II. The results show that the developed base calling scheme is significantly more computationally efficient than the best performing unsupervised method currently available, while achieving the same accuracy.

**Conclusions:**

The proposed ParticleCall provides more accurate calls than the Illumina’s base calling algorithm, Bustard. At the same time, ParticleCall is significantly more computationally efficient than other recent schemes with similar performance, rendering it more feasible for high-throughput sequencing data analysis. Improvement of base calling accuracy will have immediate beneficial effects on the performance of downstream applications such as SNP and genotype calling.

ParticleCall is freely available at https://sourceforge.net/projects/particlecall.

## Background

The advancements of next-generation sequencing technologies have enabled inexpensive and rapid generation of vast amounts of sequencing data [1-3]. At the same time, high-throughput sequencing technologies present us with the challenge of processing and analyzing large data sets that they provide. A fundamental computational challenge encountered in next-generation sequencing systems is the one of determining the order of nucleotides from the acquired measurements, the task typically referred to as *base calling*. The accuracy of base calling is of essential importance for various downstream applications including sequence assembly, SNP calling, and genotype calling [4]. Moreover, improving base calling accuracy may enable achieving desired performance of downstream applications with smaller sequencing coverage, which translates to a reduction in the sequencing cost.

A widely used sequencing-by-synthesis platform, commercialized by Illumina, relies on reversible terminator chemistry. Illumina’s sequencing platforms are supported by a commercial base-calling algorithm called Bustard. While Bustard is computationally very efficient, its base-calling error rates can be significantly improved by various computationally more demanding schemes [5]. Such schemes include work presented in [6-9]. Among the proposed methods, the BayesCall algorithm [8] has been shown to significantly outperform Bustard in terms of the achievable base calling error rates. By relying on a full parametric model of the acquired signal, BayesCall builds a Bayesian inference framework capable of providing valuable probabilistic information that can be used in downstream applications. However, its performance gains come at high computational costs. A modified version of the BaseCall algorithm named naiveBayesCall [9] performs base calling in a much more efficient way, but its accuracy deteriorates (albeit remains better than Bustard’s). Both BayesCall and naiveBayesCall rely on expectation-maximization (EM) framework that employs a Markov chain Monte Carlo (MCMC) sampling strategy to estimate the parameters of the statistical model describing the signal acquisition process. This parameter estimation step turns out to be very time-consuming, limiting practical feasibility of the proposed schemes. Highly accurate and practically feasible parameter estimation and base-calling remain a challenge that needs to be addressed.

In this paper, we propose a Hidden Markov Model (HMM) representation of the signal acquired by Illumina’s sequencing-by-synthesis platforms and develop a particle filtering (i.e., sequential Monte Carlo) base-calling scheme that we refer to as ParticleCall. When relying on the BayesCall’s Markov Chain Monte Carlo implementation of the EM algorithm (MCEM) to estimate system parameters, ParticleCall achieves the same error rate performance as BayesCall while reducing the time needed for base calling by a factor of 3. To improve the speed of parameter estimation, we develop a particle filter implementation of the EM algorithm (PFEM). PFEM significantly reduces parameter estimation time while leading to a very minor deterioration of the accuracy of base calling. Finally, we demonstrate that ParticleCall has the best discrimination ability among all of the considered base calling schemes.

## Methods

In this section, we first review the data acquisition process and the basic mathematical model of the Illumina’s sequencing-by-synthesis platform. Then we introduce a Hidden Markov Model (HMM) representation of the acquired signals. Relying on the HMM model and particle filtering (i.e., sequential Monte Carlo) techniques, we develop a novel base calling and parameter estimation scheme and discuss some important practical aspects of the proposed method.

### Illumina sequencing platform

A sequencing task on the Illumina’s platform is preceded by the preparation of a library of single-stranded short templates created by performing random fragmentation of the target DNA sample. Each single-stranded fragment in the library is placed on a glass surface (i.e., the flow cell [10]) and subjected to bridge amplification in order to create a cluster of identical copies of DNA templates [11]. The flow cell contains eight *lanes* where each lane is divided into a hundred of nonoverlapping *tiles*. The order of nucleotides in a DNA template is identified by synthesizing its complementary strand while relying on reversible terminator chemistry [3]. Ideally, in every sequencing cycle, a single fluorescently labeled nucleotide is incorporated into the complementary strand on each copy of the template in a cluster. The incorporated nucleotide is a Watson-Crick complement of the first unpaired base of the template. In reversible terminator chemistry, four distinct fluorescent tags are used to label the four bases, and are detected by CCD imaging technology. The acquired images are processed in order to obtain intensity signals indicating the type of nucleotide incorporated in each cycle. These raw signal intensities are then analyzed by a base-calling algorithm to infer the order of nucleotides in each of the templates.

Quality of the acquired raw signals is adversely affected by the imperfections in the underlying sequencing-by-synthesis and signal acquisition processes. The imperfections are manifested as various sources of uncertainties. For instance, a small fraction of the strands being synthesized may fail to incorporate a base, or they may incorporate multiple bases in a single test cycle. These effects are referred to as phasing and pre-phasing, respectively, and they result in an incoherent addition of the signals generated by the synthesis of the complementary strands on the copies of the template. Other sources of uncertainty are due to cross-talk and delay effects in the optical detection process, the residual effects that are readily observed between subsequent test cycles, signal decay, and measurement noise.

### Overview of the mathematical model

To describe the signal acquired by the Illumina’s sequencing-by-synthesis platform, a parametric model was proposed in [8]. Basic components of the model are overviewed below.

A length-*L* DNA template sequence is represented by a 4×*L* matrix *S*, where the ^*ith*^column of *S*, _**s***i*_, is considered to be a randomly generated unit vector with a single non-zero entry indicating the type of the ^*ith*^base in the sequence. We follow the convention where the first component of the vector _**s***i*_corresponds to the base A, the second to C, the third to G, and the fourth to T and denote them as _**e***A*_,_**e***C*_,_**e***G*_,_**e***T*_. The goal of base-calling is to infer unknown *S* from the signals obtained by optically detecting nucleotides incorporated during the sequencing-by-synthesis process.

Let *p* denote the average fraction of strands that fail to extend in a test cycle. Phasing is modeled as a Bernoulli random variable with probability *p*. Let *q* denote the average fraction of strands which extend by more than one base in a single test cycle. Pre-phasing is modeled as a Bernoulli random variable with probability *q*. Length of the synthesized strand changes from *i* to *j* with probability

(1) $$P_{\mathrm{ij}}=\left\{\begin{array}{ll} p, & \text{if}\;j=i,\\ 1-p-q, & \text{if}\;j=i+1,\\ q, & \text{if}\;j=i+2,\\ 0, & \text{otherwise.} \end{array}\right)$$

Let *P* denote an (*L* + 1)×(*L* + 1) transition matrix with entries _*Pij*_defined above, 1≤*i*,*j*≤*L* + 1. The signal generated over *L* cycles of the synthesis process is affected by phasing and pre-phasing and can be expressed as *X*=*SH*, where *H*=(_*Hi*,*j*_) is an *L*×*L* matrix with entries $H_{i,j}={\left[P^{j}\right]}_{1(i+1)}$, the probability that a synthesized strand is of length *i* after *j* cycles. Here ^*Pj*^ denotes the ^*jth*^ power of matrix *P*. The decay in signal intensities over cycles (caused by DNA loss due to primer-template melting, digestion by enzymatic impurities, DNA dissociation, misincorporation, etc.) is modeled by the per-cluster density random parameter _*λt*_,

(2) $$\lambda_{t}=(1-d_{t})\lambda_{t-1}+(1-d_{t})\lambda_{t-1}{𝜖}_{t},$$

where ${𝜖}_{t}∼𝒩(0,\sigma_{t}^{2})$ is a one-dimensional Gaussian random variable and _*dt*_ is the per-cluster density decay parameter within [0,1]. We represent the ^*tth*^column of *H* as _**h***t*_and the ^*tth*^ column of *X* as _**x***t*_. Incorporating the decay into the model, the signal generated in cycle *t* is expressed as

(3) $$x_{t}=\lambda_{t}Sh_{t},$$

where $x_{t}={\left[x_{t}^{A}x_{t}^{C}x_{t}^{G}x_{t}^{T}\right]}^{\prime \prime }$ is the vector of signals generated in each of the optical channels. Assuming Gaussian observation noise, the measured intensities at cycle *t* are given by

(4) $$y_{t}=K_{t}x_{t}+\underset{b\in \{A,C,G,T\}}{\sum }x_{t}^{b}\eta_{t}^{b},$$

where _*Kt*_denotes the 4×4 crosstalk matrix describing overlap of the emission spectra of the four fluorescent tags, and $\eta_{t}^{A},\eta_{t}^{C},\eta_{t}^{G},\eta_{t}^{T}$ are independent, identically distributed (i.i.d.) 4×1 Gaussian random vectors with zero mean and a common 4×4 covariance matrix _*Σt*_.

Note that, due to typically small values of *p* and *q*, the components of the vector _**h***t*_ around its ^*tth*^ entry are significantly greater than the remaining ones. This observation can be used to simplify the expressions (2) and (3). In particular, let $h_{t}^{w}$ denote the vector obtained by windowing _**h***t*_ around its ^*tth*^ entry, i.e., by setting small components of _**h***t*_ to 0. In general, we consider *l* + *r* + 1 dominant components of _**h***t*_centered at position *t*, _*Ht*−*l*,*t*_,_*Ht*−*l* + 1,*t*_,…,_*Ht*,*t*_,…_*Ht* + *r*−1,*t*_,_*Ht* + *r*,*t*_, and then expression (2) becomes

(5) $$x_{t}\approx x_{t}^{w}=\lambda_{t}Sh_{t}^{w}=\lambda_{t}\overset{r}{\underset{i=-l}{\sum }}s_{t+i}H_{t+i,t}.$$

Finally, note that the signal measured in cycle *t* is empirically observed to contain residual effect from the previous cycle. The residual effect is modeled by adding _*αt*_(1−_*dt*_)_**y***t*−1_to _**y***t*_, where the unknown parameter _*αt*_∈(0,1). Therefore, the model can be summarized as

(6) $$\begin{array}{ll} \lambda_{t}|\lambda_{t-1}∼ & 𝒩\left((1-d_{t})\lambda_{t-1},{\left(1-d_{t}\right)}^{2}\lambda_{t-1}^{2}\sigma_{t}^{2}\right),\\ y_{t}|y_{t-1},S,\lambda_{t}∼ & 𝒩\left(K_{t}x_{t}^{w}+\alpha_{t}(1-d_{t})y_{t-1},∥x_{t}^{w}{∥}_{2}^{2}\Sigma_{t}\right),\\ s_{t}∼ & \mathrm{Unif}\left(e_{A},e_{C},e_{G},e_{T}\right),\\ x_{t}^{w}= & \lambda_{t}Sh_{t}^{w}\\ t=1,2,\ldots ,L \end{array}$$

where ∥·_∥2_ denotes the _*l*2_-norm of its argument, and where _**y**0_=**0**, _*λ*0_=1.

### Hidden Markov Model of DNA base-calling

In this section, we reformulate the statistical description of the signal acquired by the Illumina’s sequencing-by-synthesis platform as a Hidden Markov Model (HMM) [12]. HMMs comprise a family of probabilistic graphical models which describe a series of observations by a “hidden” stochastic process and are generally suitable for representing time series data. Sequencing data obtained from the Illumina’s platform is a set of time-series intensities _**y**1:*L*_, motivating the HMM representation. HMMs provide a convenient framework for state and parameter estimation, which we exploit to develop a particle filter base-calling scheme in the next section.

For the sake of convenience, we remove the dependency between subsequent observations _**y***t*−1_ and _**y***t*_ by defining $y_{t}^{{}^{\prime \prime }}=y_{t}-\alpha_{t}(1-d_{t})y_{t-1},t=1,2,\ldots ,L$. Therefore, we can write

(7) $$y_{t}^{{}^{\prime \prime }}|S,\lambda_{t}∼𝒩\left(K_{t}x_{t}^{w},∥x_{t}^{w}{∥}_{2}^{2}\Sigma_{t}\right).$$

Components of $y_{1:L}^{{}^{\prime \prime }}$ are the observations of our HMM, and depend on the underlying signals _**x**1:*L*_. Moreover, let $S_{t}^{w}$ denote the 4×(*l* + *r* + 1) windowed submatrix of *S*, i.e.,

(8) $$S_{t}^{w}=[s_{t-l}s_{t-l+1}\ldots s_{t}\ldots s_{t+r}].$$

Since $x_{t}^{w}=\lambda_{t}Sh_{t}^{w}=\lambda_{t}\overset{t+r}{\underset{i=t-l}{\sum }}s_{i}H_{i,t}$, it is clear that $y_{t}^{{}^{\prime \prime }}$ depends on _*λt*_and $S_{t}^{w}$. Therefore, we define the state of the HMM to be the combination of _*λt*_ and $S_{t}^{w}$ – the per-cluster density at cycle *t* and the collection of (*l* + *r* + 1) bases around (and including) the base in position *t*, respectively.

**Figure 1:**
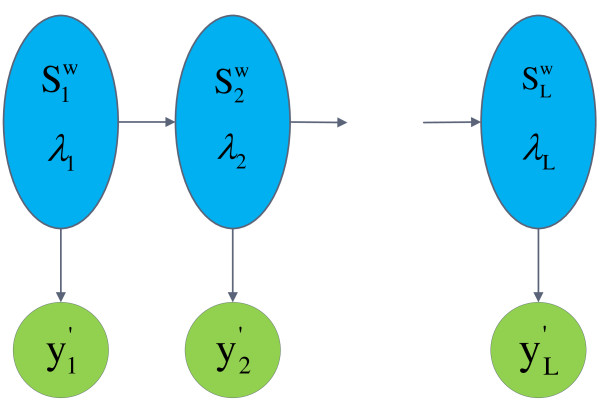
**A hidden Markov model of the generated signal in Illumina sequencing-by-synthesis platforms.** An illustration of the graphical HMM of the Illumina’s sequencing platform. The observations ^**y***″*^represent signal intensities after the removal of residual effects. The states are the combinations of $S_{t}^{w}$ and _*λt*_, which represent a subsequence of the template centered at position *t* and per-cluster density, respectively.

The proposed HMM representation is illustrated in Figure 1. The observation dynamics that characterize the relationship between $y_{t}^{{}^{\prime \prime }}$ and the hidden states $\left(S_{t}^{w},\lambda_{t}\right)$ are given by the distribution $g(y_{t}^{{}^{\prime \prime }}|S_{t}^{w},\lambda_{t})$. It is straightforward to show from (5) that

(9) $$g\left(y_{t}^{{}^{\prime \prime }}|S_{t}^{w},\lambda_{t}\right)∼𝒩\left(K_{t}x_{t}^{w},∥x_{t}^{w}{∥}_{2}^{2}\Sigma_{t}\right).$$

On the other hand, the state transition dynamics is described by the transition probability between subsequent states, $\left(S_{t-1}^{w},\lambda_{t-1}\right)$ and $\left(S_{t}^{w},\lambda_{t}\right)$. Since $S_{t}^{w}$ and _*λt*_ are independent, the transition probability is

(10) $$f(S_{t}^{w},\lambda_{t}|S_{t-1}^{w},\lambda_{t-1})=f_{1}(S_{t}^{w}|S_{t-1}^{w})f_{2}(\lambda_{t}|\lambda_{t-1}).$$

The second term on the right-hand side of (8), _*f*2_(_*λt*_|_*λt*−1_), is known from the density decay model (1),

(11) $$f_{2}(\lambda_{t}|\lambda_{t-1})∼𝒩((1-d_{t})\lambda_{t-1},{\left(1-d_{t}\right)}^{2}\lambda_{t-1}^{2}\sigma_{t}^{2}).$$

For notational convenience, we use $s_{t,1}^{w},\ldots ,s_{t,l+r+1}^{w}$ to denote the set of *l* + *r* + 1 column vectors of $S_{t}^{w}$. Note that for *k*=2,3,…,*l* + *r* + 1, the column vectors $s_{t-1,k}^{w}$ in $S_{t-1}^{w}$ and the column vectors $s_{t,k-1}^{w}$ in $S_{t}^{w}$ actually represent the same base. Therefore, the transition model between them can be represent by a *δ* function as

(12) $$\begin{array}{lcr} p(s_{t,k-1}^{w}|s_{t-1,k}^{w}) & = & \delta_{\left\{s_{t,k-1}^{w}=s_{t-1,k}^{w}\right\}}\\ & = & \left\{\begin{array}{ll} 1, & \;\text{if}s_{t,k-1}^{w}=s_{t-1,k}^{w},\\ 0, & \;\text{if}s_{t,k-1}^{w}\neq s_{t-1,k}^{w}. \end{array}\right) \end{array}$$

Let *U*({_**e***A*_,_**e***C*_,_**e***G*_,_**e***T*_}) denote a uniform distribution on the support set of unit vectors ({_**e***A*_,_**e***C*_,_**e***G*_,_**e***T*_}). We assume no correlation between consecutive bases of the template sequence, i.e., $s_{t,l+r+1}^{w}$ is generated from *U*({_**e***A*_,_**e***C*_,_**e***G*_,_**e***T*_}). Therefore, $f_{1}(S_{t}^{w}|S_{t-1}^{w})$ can be written as

(13) $$f_{1}(S_{t}^{w}|S_{t-1}^{w})=\left(\overset{l+r+1}{\underset{k=2}{\prod }}\delta_{\left\{s_{t-1,k}^{w}=s_{t,k-1}^{w}\right\}}\right)u\left(s_{t,l+r+1}^{w}\right),$$

where *u*(·)∼*U*({_**e***A*_,_**e***C*_,_**e***G*_,_**e***T*_}). Hereby, all the components of the HMM are specified.

### ParticleCall base-calling algorithm

The goal of base calling is to determine the order of nucleotides in a template from the acquired signal _**y**1:*t*_. This can be rephrased as the problem of inferring the most likely sequence of states $\left(S_{t}^{w},\lambda_{t}\right)$ of the HMM in (7)-( 8) from the observed sequence $y_{1:t}^{\prime \prime }$(clearly, _**s**1:*L*_ follows directly from $S_{t}^{w}$). We assume that the parameters *Λ*={*p*,*q*,_*d*1:*L*_,_*α*1:*L*_,_*σ*1:*L*_,_*K*1:*L*_,_*Σ*1:*L*_} are common for all clusters within a tile, and that they are provided by a parameter estimation step discussed in the following section. In this section, we introduce a novel base calling algorithm ParticleCall which relies on particle filtering techniques to sequentially infer $\left(S_{t}^{w},\lambda_{t}\right)$ and, therefore, recover the matrix *S*.

In general, particle filtering (i.e., sequential Monte Carlo) methods generate a set of particles with associated weights to estimate the posteriori distribution of unknown variables given the acquired measurements [13]. In the proposed HMM framework, we sequentially calculate the posteriori distribution of the columns of *Sp*(**s**_*t*_|$y_{1:t}^{\prime \prime }$),*t*=1,2,…,*L*, and find the maximum a posteriori (MAP) estimates of _**s***t*_ by solving

(14) $${\hat{s}}_{t}=\arg\underset{s_{t}\in \{e_{A},e_{C},e_{G},e_{T}\}}{\max}\{p(s_{t}|\overset{\prime \prime }{\underset{1:t}{y}})\}.$$

Our algorithm relies on a sequential importance sampling/resampling (SISR) particle filter scheme [14] to calculate $p(S_{t}^{w},\lambda_{t}|y_{1:t}^{\prime \prime })$. Different choices and approximation methods of proposal densities are considered in [15-17]. We directly use the transition (8) as the proposal density. This sequential importance sampling suffers from degeneracy and the variance of the importance weights will increase over time. To address the degeneracy problem, a resampling step is introduced in order to eliminate samples which have small normalized importance weights. Common resampling methods include multinomial resampling [14], residual resampling [18] and systematic resampling [19,20]. We measure degeneracy of the algorithm using the effective sample size _*K*eff_ and, for the sake of simplicity, employ multinomial resampling strategy. If we denote the number of particles by _*Np*_ and associated weights by *w*, then $K_{\text{eff}}={\left(\overset{N_{p}}{\underset{k=1}{\sum }}{\left(w_{t}^{\left(i\right)}\right)}^{2}\right)}^{-1}$ and resampling step is used when _*K*eff_ is below a fixed threshold _*Nthreshold*_. _*Nthreshold*_ of size *O*(_*Np*_) is typically sufficient [14]. In our implementation, we set _*Nthreshold*_=_*Np*_/2.

We omit further details for brevity and formalize the ParticleCall algorithm below.

#### 

##### Algorithm 1

ParticleCall base-calling algorithm 

1. Initialization:

1.1 Initialize particles:**for***i*=1→_*Np*_**do**

 Sample each column of the submatrix $S_{1}^{w,(i)}$ from *U*({_**e***A*_,_**e***C*_,_**e***G*_,_**e***T*_}); Sample $\lambda_{1}^{\left(i\right)}$ from a Gaussian distribution with mean 1, and the variance calculated using Bustard’s estimates of *λ*in the first 10 test cycles.**end for**

1.2 Compute and normalize weights for each particle according to $w_{1}^{\left(i\right)}\propto g(y_{1}^{\prime \prime }|S_{1}^{w,(i)},\lambda_{1}^{\left(i\right)})$ as in (7).

2. Run iteration *t*(*t*≥2):

2.1 Sampling:**for***i*=1→_*Np*_**do**

 Sample $S_{t}^{w,(i)},\lambda_{t}^{\left(i\right)}∼f(\cdot ,\cdot |S_{t-1}^{w,(i)},\lambda_{t-1}^{\left(i\right)})$ according to (8).**end for**

2.2 Update the importance weight

(15) $$\begin{array}{lcr} w_{t}^{\left(i\right)}\propto w_{t-1}^{\left(i\right)}g\left(y_{t}^{\prime \prime }|S_{t}^{w,(i)},\lambda_{t}^{\left(i\right)}\right). & & \end{array}$$

2.3 Normalize the weights. Calculate the posteriori probability of _**s***t*_ and obtain the estimate ${\hat{s}}_{t}$.

2.4 Resampling:**if**$K_{\text{eff}}={\left(\overset{N_{p}}{\underset{k=1}{\sum }}{\left(w_{t}^{\left(i\right)}\right)}^{2}\right)}^{-1}\leq N_{\text{threshold}}$**then**

 Draw _*Np*_samples $\left\{{\bar{S}}_{t}^{w,(j)},{\bar{\lambda }}_{t}^{\left(j\right)},j=1,\ldots ,N_{p}\right\}$ from $\left\{S_{t}^{w,(i)},\lambda_{t}^{\left(i\right)},i=1,\ldots ,N_{p}\right\}$ with probabilities proportional to $\left\{w_{t}^{\left(i\right)},i=1,\ldots ,N_{p}\right\}$. Assign equal weight to each particle, ${\bar{w}}_{t}^{\left(i\right)}=1/N_{p}$.**end if**

Since $S_{t}^{w}$ in the HMM states are discrete with a finite alphabet, and the transitions of $S_{t}^{w}$ and _*λt*_ are independent according to (8), it is possible to Rao-Blackwellize the ParticleCall algorithm. Rao-Blackwellization is used to marginalize part of the states in the particle filter, hence reducing the number of needed particles _*Np*_[16]. We marginalize the discrete states $S_{t}^{w}$ and reduce the hidden process to _*λt*_, while relying on the particle filter to calculate *p*(_*λ*1:*t*_|$y_{1:t}^{\prime \prime }$).

The original posterior distribution of the states can be expressed as

(16) $$p\left(\lambda_{1:t},S_{1:t}^{w}|y_{1:t}^{\prime \prime }\right)=p\left(S_{1:t}^{w}|y_{1:t}^{\prime \prime },\lambda_{1:t}\right)p\left(\lambda_{1:t}|y_{1:t}^{\prime \prime }\right).$$

Since $p(\lambda_{1:t}|y_{1:t}^{\prime \prime })\propto p(y_{t}^{\prime \prime }|y_{1:t-1}^{\prime \prime },\lambda_{1:t})p(\lambda_{t}|\lambda_{t-1}^{\left(i\right)})$, where $\lambda_{t-1}^{\left(i\right)}$ is a sample from *p*(_*λ*1:*t*−1_|$y_{1:t-1}^{\prime \prime }$), we can state the Rao-Blackwellized ParticleCall algorithm as below.

##### Algorithm 2

Rao-Blackwellized ParticleCall algorithm 

1. Initialization:

1.1 Initialize particles:**for***i*=1→_*Np*_**do**

 Sample $\lambda_{1}^{\left(i\right)}$ from a Gaussian distribution with mean 1, and the variance calculated using Bustard’s estimates of *λ*in the first 10 test cycles.**end for**

1.2 Compute and normalize weights for each particle according to $w_{1}^{\left(i\right)}\propto g(y_{1}^{\prime \prime }|\lambda_{1}^{\left(i\right)})\propto \underset{S_{1}^{w}}{\sum }g(y_{1}^{\prime \prime }|S_{1}^{w},\lambda_{1}^{\left(i\right)})$.

1.3 Calculate the discrete distribution $p(S_{1}^{w}|y_{1},\lambda_{1}^{\left(i\right)})$ for each *i*.

2. Run iteration *t*(*t*≥2):

2.1 Sampling:**for***i*=1→_*Np*_**do**

 Sample $\lambda_{t}^{\left(i\right)}∼f(\cdot |\lambda_{t-1}^{\left(i\right)})$.**end for**

2.2 Update the importance weight $w_{t}^{\left(i\right)}\propto w_{t-1}^{\left(i\right)}g(y_{t}^{\prime \prime }|y_{1:t-1}^{\prime \prime },\lambda_{1:t}^{\left(i\right)}).$ and normalize the weights.

2.3 Resample if _*K*eff_≤_*Nthreshold*_

2.4 Update $p(S_{t}^{w}|y_{1:t}^{\prime \prime },\lambda_{1:t}^{\left(i\right)})$**for***i*=1→_*Np*_**do**

 Update $p(S_{t}^{w}|y_{1:t}^{\prime \prime },\lambda_{1:t}^{\left(i\right)})$ using $p(S_{t-1}^{w}|y_{1:t-1}^{\prime \prime },\lambda_{1:t-1}^{\left(i\right)})$ and $\lambda_{t}^{\left(i\right)}$.**end for**

In step 2.2 of Algorithm 2, the quantity $g(y_{t}^{\prime \prime }|y_{1:t-1}^{\prime \prime },\lambda_{1:t}^{\left(i\right)})$ can be obtained by marginalizing over discrete states $S_{t}^{w}$ and $S_{t-1}^{w}$,

(17) $$\begin{array}{c} g\left(y_{t}^{\prime \prime }|y_{1:t-1}^{\prime \prime },\lambda_{1:t}^{\left(i\right)}\right)\\ =\underset{S_{t}^{w}}{\sum }p\left(y_{t}^{\prime \prime }|y_{1:t-1}^{\prime \prime },S_{t}^{w},\lambda_{1:t}^{\left(i\right)}\right)p\left(S_{t}^{w}|y_{1:t-1}^{\prime \prime },\lambda_{1:t}^{\left(i\right)}\right)\\ =\underset{S_{t}^{w}}{\sum }p\left(y_{t}^{\prime \prime }|S_{t}^{w},\lambda_{t}^{\left(i\right)}\right)\underset{S_{t-1}^{w}}{\sum }\left[p\left(S_{t}^{w}|S_{t-1}^{w},y_{1:t-1}^{\prime \prime },\lambda_{1:t}^{\left(i\right)}\right)\right)\\ \;\left(\times p\left(S_{t-1}^{w}|y_{1:t-1}^{\prime \prime },\lambda_{1:t}^{\left(i\right)}\right)\right], \end{array}$$

where $p(y_{t}^{\prime \prime }|S_{t}^{w},\lambda_{t}^{\left(i\right)})$ is the observation density, $p(S_{t-1}^{w}|$$y_{1:t-1}^{\prime \prime },\lambda_{1:t}^{\left(i\right)})=p(S_{t-1}^{w}|y_{1:t-1}^{\prime \prime },\lambda_{1:t-1}^{\left(i\right)})$ due to the independence of the state transitions, and $p(S_{t}^{w}|S_{t-1}^{w},y_{1:t-1}^{\prime \prime },\lambda_{1:t}^{\left(i\right)})=$$p(S_{t}^{w}|S_{t-1}^{w})$ due to the Markov property and the independence of the state transitions.

In step 2.4 of Algorithm 2, the update equation is obtained as

(18) $$\begin{array}{lll} & p\left(S_{t}^{w}|y_{1:t}^{\prime \prime },\lambda_{1:t}^{\left(i\right)}\right)\propto p\left(S_{t}^{w},y_{t}^{\prime \prime },\lambda_{t}^{\left(i\right)}|y_{1:t-1}^{\prime \prime },\lambda_{1:t-1}^{\left(i\right)}\right)\; & \;\\ & =\underset{S_{t-1}^{w}}{\sum }\left[p\left(y_{t}^{\prime \prime },S_{t}^{w},\lambda_{t}^{\left(i\right)}|y_{1:t-1}^{\prime \prime },S_{t-1}^{w},\lambda_{1:t-1}^{\left(i\right)}\right)\right)\; & \;\\ & \;\left(\times p\left(S_{t-1}^{w}|y_{1:t-1}^{\prime \prime },\lambda_{1:t-1}^{\left(i\right)}\right)\right]\; & \;\\ & =\underset{S_{t-1}^{w}}{\sum }\left[p\left(y_{t}^{\prime \prime }|S_{t}^{w},\lambda_{t}^{\left(i\right)},y_{1:t-1}^{\prime \prime },S_{t-1}^{w},\lambda_{1:t-1}^{\left(i\right)}\right)\right)\; & \;\\ & \;\times p\left(S_{t}^{w},\lambda_{t}^{\left(i\right)}|y_{1:t-1}^{\prime \prime },S_{t-1}^{w},\lambda_{1:t-1}^{\left(i\right)}\right)\; & \;\\ & \;\left(\times p\left(S_{t-1}^{w}|y_{1:t-1}^{\prime \prime },\lambda_{1:t-1}^{\left(i\right)}\right)\right]\; & \;\\ & =p\left(y_{t}^{\prime \prime }|S_{t}^{w},\lambda_{t}^{\left(i\right)}\right)p\left(\lambda_{t}^{\left(i\right)}|\lambda_{t-1}^{\left(i\right)}\right)\; & \;\\ & \;\times \underset{S_{t-1}^{w}}{\sum }p\left(S_{t}^{w}|S_{t-1}^{w}\right)p\left(S_{t-1}^{w}|y_{1:t-1}^{\prime \prime },\lambda_{1:t-1}^{\left(i\right)}\right)\; \end{array}$$

### Parameter estimation

To determine the set of parameters *Λ*needed to run the proposed ParticleCall base calling algorithm, one could rely on the MCMC implementation of the EM algorithm (MCEM) proposed in [8]. In section Results and discussion, we demonstrate the performance of the ParticleCall algorithm that relies on the MCEM parameter estimation scheme. Note, however, that the MCMC sampling strategy employed by MCEM requires a lengthy burn-in period and a very large sample size to perform the expectation step. Therefore, the MCEM parameter estimation scheme is computationally rather intensive and requires significant computational resources if it is to be used for processing large sequencing data sets. As an alternative, we develop an EM parameter estimation scheme which relies on the proposed HMM and uses samples generated by a particle filter to evaluate the expectation of the likelihood function. We refer to this algorithm as the particle filter EM (PFEM). The speed and accuracy of the proposed scheme is practically sound for use in next generation sequencing platforms.

#### Assumptions on parameters

Recall that the set of parameters needed to run ParticleCall is *Λ*={*pq*_*d*1:*L*__*α*1:*L*__*σ*1:*L*__*K*1:*L*__*Σ*1:*L*_}. The phasing and prephasing parameters *p* and *q* are assumed to be the same for each sequencing lane and are estimated using the same procedure as Bustard (see, e.g., [8]). The remaining parameters are assumed to be cycle-dependent and need to be estimated for each tile. The cycle-dependency assumption on the parameters can lead to a substantial improvement in the base-calling accuracy [5]. In order to avoid over-fitting, we assume that parameters remain constant within a short window of cycles and then change to a different set of values. To track the changes in the parameters, we first divide the total read length *L* into several non-overlapping windows and then perform our parameter estimation window-by-window. To further reduce the number of parameters and improve the estimation efficiency, we assume that the parameters _*d*1:*L*_ and _*σ*1:*L*_ are uniformly distributed over an interval and incorporate them into the hidden states of the HMM model. Therefore, only the mean and variance of these parameters, i.e., _*dmean*_, _*dvar*_, _*σmean*_, and _*σvar*_need to be estimated. Computational results demonstrate that these two assumptions does not affect the accuracy of base-calling.

#### Particle filter EM algorithm

In the early sequencing cycles, effects of phasing and prephasing are relatively small. Therefore, we may ignore phasing and prephasing to facilitate straight-forward computation of the initial estimates of the remaining parameters. In particular, the signal generated in the early cycles *t* is approximated as

(19) $$x_{t}=\lambda_{t}s_{t}.$$

Replacing (2) by (10) leads to a simplified model that allows for straightforward base calling and inference of the parameters by means of linear regression. We use these values to obtain the estimates of _*dmean*_, _*dvar*_, _*σmean*_, and _*σvar*_, and to initialize the remaining parameters *α*, *K*, *Σ*, in the particle filter EM parameter estimation procedure.

The parameter estimation is performed window-by-window and is conducted using *n* reads randomly chosen from a tile (in our experiments, we use *n*=200). Assume the window length is *w*, and denote the window index by *m*. The particle filter EM (PFEM) algorithm finds parameters for one window and then uses these values to initialize the search for parameters in the next window. We illustrate the procedure for the first window here (the same procedure is repeated in the following windows). Let $\Lambda_{1}^{i}=\{\alpha^{i},K^{i},\Sigma^{i}\}$ denote the set of parameters for window 1 in the *i*th iteration of the EM scheme. The estimate of $\Lambda_{1}^{i}$ is given by

(20) $$\Lambda_{1}^{i}=\arg\underset{\Lambda_{1}}{\max}L_{1}\left(\overset{i-1}{\underset{1}{\Lambda }}\right),$$

where $L_{1}(\Lambda_{1}^{i-1})=\overset{n}{\underset{j=1}{\sum }}L_{1,j}(\Lambda_{1}^{i-1})$ is the sum of the log-likelihood functions over the reads in the training set. The log-likelihood function for each read, $L_{1,j}(\Lambda_{1}^{i-1})$, is obtained as

(21) $$\begin{array}{lll} L_{1,j}(\Lambda_{1}^{i-1}) & =\log P(y_{1:w}|\Lambda_{1}^{i-1})\; & \;\\ & =E\left[\log P(y_{1:w},s_{1:w},\lambda_{1:w}|\Lambda_{1}^{i-1})\right],\; \end{array}$$

where the expectation is taken with respect to $P(s_{1:w},\lambda_{1:w}|y_{1:w},\Lambda_{1}^{i-1})$. We rely on an SISR particle filtering scheme to generate equally weighted sample trajectories from $P(s_{1:w},\lambda_{1:w}|y_{1:w},\Lambda_{1}^{i-1})$. Based on (7) and (8), we calculate $\log P(y_{1:w},s_{1:w},\lambda_{1:w}|\Lambda_{1}^{i-1})$ for these samples and compute their average to approximate the expectation in (12). The maximization (11) is performed by solving equations obtained after taking gradients of $L_{1}(\Lambda_{1}^{i-1})$ over the parameters and setting them to 0. In our experiment, the PFEM parameter estimation scheme performs 30 EM iterations and uses 600 samples from the particle filter for each window.

## Results and discussion

The proposed method is evaluated on a data set obtained by sequencing phiX174 bacteriophage using Illumina Genome Analyzer II with the cycle length 76. This is a short genome with a known sequence which enables reliable performance comparison of different base-calling techniques. We tested ParticleCall and several other algorithms on a tile containing 77337 reads, and present the results here. All the codes are written in C and the tests are run on a desktop with an Intel Core i7 4-core 3GHz processor.

**Figure 2:**
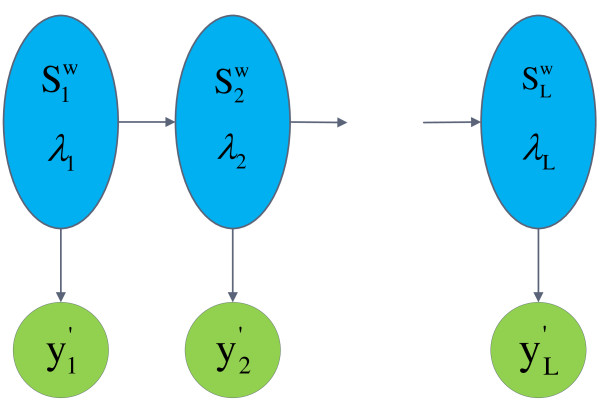
**Per-cycle error rates of ParticleCall, BayesCall, naiveBayesCall, Rolexa and Bustard.** The figure compares the per-cycle error rates of different base-calling algorithms. ParticleCall and BayesCall are the most accurate ones.

### Performance of ParticleCall

The base calling error rates are computed by aligning the reads to the reference genome and evaluating frequency of mismatches. Reads that could not be aligned to the reference with at least 70% matches are discarded. Note that the error rates and speed of the proposed ParticleCall algorithm and the parameter estimation scheme are affected by the parameters *l*, *r*, particle number _*Np*_, and parameter estimation window length *w*. We ran ParticleCall with *l*=*r*∈{1,2,4}. Increasing *l* and *r* beyond *l*=*r*=1 did not affect the performance while it significantly slowed down the algorithm. This is due to small values of the phasing and prephasing probabilities, which are estimated to be *p*=3.54×1^0−8^ and *q*=0.00335. Therefore, in the remainder of the paper, we set *l*=*r*=1. The accuracy of base-calling for different _*Np*_is shown in Table 1. As seen there, for the original ParticleCall algorithm, _*Np*_=800 leads to high performance with reasonable speed. Rao-Blackwellized ParticleCall can achieve the same accuracy with fewer particles (in particular, _*Np*_=300); however, its effective running time is 3 times that of the original ParticleCall with the same performance. This is because the Rao-Blackwellization steps in (9) and (9) require evaluating a sum over all possible $S_{t}^{w}$ (4^3^=64 for our choice *l*=*r*=1), resulting in a fairly large number of basic operations needed to calculate exact distribution of the discrete variables. Therefore, for further performance comparisons, we rely on the original ParticleCall algorithm (formalized as Algorithm 1). Table 2 shows the ParticleCall base calling error rate and parameter estimation times for different window lengths *w*. In the remainder of the paper, we set *w*=5 as it leads to desirable performance/speed characteristics of the algorithm.

**Table 1:** **Comparison of ParticleCall with different**_*N****p***_

Method	_*Np*_	error rate	base-calling time (min)	
ParticleCall (via MCEM)	400	0.0126	46	
	800	0.0124	88	
	1200	0.0124	130	
ParticleCall (via PFEM)	400	0.0128	46	
	800	0.0125	91	
	1200	0.0125	133	
Rao-Blackwellized ParticleCall (via MCEM)	100	0.0128	103	
	200	0.0125	190	
	300	0.0124	287	
	400	0.0124	386	

ParticleCall is run using parameters obtained via the MCEM parameter estimation scheme as well as via the PFEM parameter estimation algorithm proposed in this paper. Rao-Blackwellized ParticleCall is run using parameters via the MCEM parameter estimation scheme.

**Table 2:** ParticleCall parameter estimation

		parameter estimation
Window length *w*	base-calling error rate	time (min)
4	0.0125	50
5	0.0125	39
6	0.0127	29
7	0.0130	25

ParticleCall base-calling error rate and the parameter estimation time of the proposed PFEM parameter estimation algorithm.

### Performance comparison of different algorithms

The error rates and speed of the proposed ParticleCall algorithm are compared with those of BayesCall, naiveBayesCall, Rolexa, and Bustard. We run ParticleCall both with parameters provided by the computationally intensive MCEM algorithm as well as with those inferred by the PFEM parameter estimation scheme proposed in this paper. The results are reported in Table 3. Note that Rolexa generally outputs the so-called IUPAC codes, unlike all the other considered algorithms which provide sequences of nucleotides A, C, G, and T. To allow a comparison, we enforce Rolexa to output sequences of nucleotides as well. The comparison of per-cycle error rates is shown in Figure 2. It can be seen from Table 3 and Figure 2 that ParticleCall, BayesCall and naiveBayesCall all have improved base-calling accuracy compared to Bustard. BayesCall is highly accurate but relatively slow – it requires approximately 4 hours to complete base-calling for one tile of the data. naiveBayesCall significantly improves base-calling speed over BayesCall but it does so at the expense of incurring higher error rate. Our ParticleCall base-calling algorithm has the same accuracy as BayesCall, while being roughly 3 times faster. Figure 2 shows that both ParticleCall and BayesCall are more accurate than naiveBayesCall in the early cycles and improve over Bustard in all cycles. Note that Bustard outperforms Rolexa, which is consistent with the results in [5]. Moreover, we see from Table 3 that performing parameter estimation via the MCEM algorithm proposed in [8] requires 19 hours, while the particle filter implementation of the EM estimation scheme proposed in this paper takes only 39 minutes. As evident from Table 3, running ParticleCall with parameters obtained by the PFEM scheme leads to only a minor performance degradation compared to running it with parameters obtained by the MCEM algorithm. Running ParticleCall base calling along with the PFEM parameter estimation scheme takes about 2 hours per tile, which is 9 times faster than the total time required by the less accurate naiveBayesCall.

**Table 3:** Comparison of error rates and speed

		base-calling	parameter estimation	
Method	error rate	time (min)	time (min)	
Bustard	0.0152	2 (total)		
Rolexa	0.0170	35 (total)		
naiveBayesCall	0.0132	21	1139	
BayesCall	0.0124	231	1139	
ParticleCall				
(via MCEM)	0.0124	88	1139	
ParticleCall				
(via PFEM)	0.0125	91	39	

The base-calling error rate and the running times of different algorithms. ParticleCall is run using parameters obtained via the MCEM parameter estimation scheme as well as via the PFEM parameter estimation algorithm proposed in this paper. For Bustard and Rolexa, only the total running times are reported.

### Quality scores

Quality scores are used to characterize confidence in the outcome of the base-calling procedures. They are computed as part of the analysis of the acquired raw data and may be used to filter out reads of suspect quality, or to shorten the reads if the quality scores of individual bases fall below certain thresholds. They can also provide confidence information for downstream analysis including sequence assembly and SNP and genotype calling. Frequently used are the so-called *phred* quality scores, which were originally developed to assess the quality of the conventional Sanger sequencing and automate large-scale sequencing projects. Phred scores are also often provided by the algorithms used for base-calling in next generation sequencing platforms. Formally, the phred score for a called base in the cycle *t*, ${\hat{s}}_{t}$, is defined as

(22) $$Q_{\mathrm{phred}}({\hat{s}}_{t})=-10\underset{10}{\log}P({\hat{s}}_{t}\neq s_{t}).$$

Essentially, $Q_{\mathrm{phred}}({\hat{s}}_{t})$ is the scaled logarithm of the error probability. Higher quality scores imply smaller probability of the base-calling error. For the proposed ParticleCall algorithm, probability of correctly calling a base can be obtained from the posteriori probability as

(23) $$P({\hat{s}}_{t}\neq s_{t})=1-p\left(s_{t}|y_{1:t}^{\prime \prime }\right).$$

**Figure 3:**
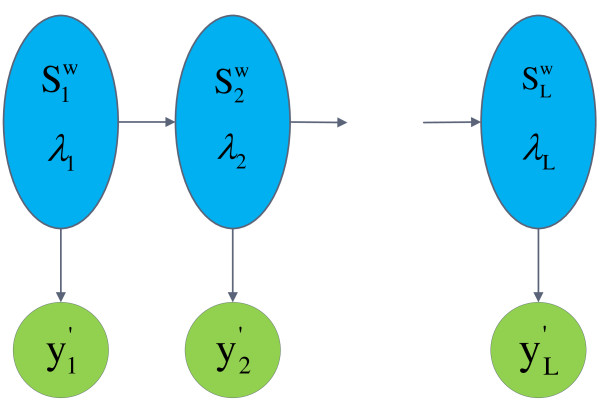
**Discrimination ability***D*(*ε***) of quality scores vs error tolerance.** The figure shows the percentage of correctly called bases under different error tolerance *ε*.

Quality scores can be used to compare the discrimination ability of different algorithms. The discrimination score *D*(*ε*) at error tolerance *ε* is defined as the ratio of the correctly called bases having $P({\hat{s}}_{t}\neq s_{t})<𝜖$ (i.e., the quality score higher than $-10\underset{10}{\log}(𝜖)$) to all called bases. Figure 3 compares the discrimination ability of ParticleCall, BayesCall, naiveBayesCall and Bustard. It shows that for a reasonable error tolerance *ε*, ParticleCall with parameters obtained through MCEM has better discrimination ability than BayesCall, naiveBayesCall and Bustard, while ParticleCall with parameters obtained through PFEM has discrimination ability close to naiveBayesCall and better than other algorithms. In other words, when a small cutoff error tolerance *ε* is set and all the bases with quality scores below *ε*are considered invalid, ParticleCall provides the most accurate results among the considered base-calling schemes.

### Effects of improved base-calling accuracy on de novo sequence assembly

In shotgun sequencing, a long target sequence is oversampled by a library of randomly fragmented copies of the target, and the overlaps between short reads obtained by a high-throughput platform are used to assemble the target. In *de novo* assembly, the target is reconstructed without consulting any reference [21,22]. Performance of assembly algorithms highly depends on the accuracy of base calling. To demonstrate the effects of base-calling accuracy on assembly, we apply the Velvet assembly algorithm [22] on reads provided by Bustard, Rolexa, naiveBayesCall, BayesCall, and ParticleCall. In particular, we randomly subsample the set of reads provided by each of the base calling algorithms to emulate 5X, 10X, 15X, and 20X coverage. Then we run Velvet on each of the subsets, and evaluate commonly used metrics that quantify the quality of the assembly procedure. Specifically, we evaluate the maximum contig length and the N50 contig length. The described procedure is repeated 200 times to obtain average values of these two quality metrics. The results are shown in Table 4. As can be seen there, ParticleCall provides the largest N50 and maximum contig length among all of the considered base calling schemes, for all of the considered coverages.

**Table 4:** de novo assembly results

									ParticleCall		ParticleCall	
Coverage	Bustard		Rolexa		naiveBayesCall		BayesCall		via MCEM		via PFEM	
	N50	Max	N50	Max	N50	Max	N50	Max	N50	Max	N50	Max
5X	271	607	259	565	278	604	292	629	299	637	289	632
10X	1169	1750	971	1557	1180	1731	1269	1831	1316	1900	1341	1865
15X	3624	3823	2885	3170	3726	3908	3466	3741	3742	3935	3697	3918
20X	4694	4744	4529	4614	4756	4816	4827	4875	5102	5116	4795	5039

The maximum contig length and N50 length of *de novo* assembly using Velvet. The average values over 200 experiments are shown in the table.

## Conclusions

In this paper we presented ParticleCall, a particle filtering algorithm for base calling in the Illumina’s sequencing-by-synthesis platform. The algorithm is developed by relying on an HMM representation of the sequencing process. Experimental results demonstrate that the ParticleCall base calling algorithm is more accurate than Bustard, Rolexa, and naiveBayesCall. It is as accurate as BayesCall while being significantly faster. Quality score analysis of the reads indicates that ParticleCall has better discrimination ability than BayesCall, naiveBayesCall and Bustard. Moreover, a novel particle filter EM (PFEM) parameter estimation scheme, much faster than the existing Monte Carlo implementation of the EM algorithm, was proposed. When relying on the PFEM scheme, ParticleCall has near-optimal performance while needing much shorter total parameter estimation and base calling time.

## Author’s contributions

Algorithms and experiments were designed by Xiaohu Shen (XS) and Haris Vikalo (HV). Algorithm code was implemented and tested by XS. The manuscript was written by XS and HV. Both authors read and approved the final manuscript.

## Competing interests

The authors declare that they have no competing interests.
